# Everolimus is better than rapamycin in attenuating neuroinflammation in kainic acid-induced seizures

**DOI:** 10.1186/s12974-017-0797-6

**Published:** 2017-01-21

**Authors:** Ming-Tao Yang, Yi-Chin Lin, Whae-Hong Ho, Chao-Lin Liu, Wang-Tso Lee

**Affiliations:** 1 Department of Pediatrics, Far Eastern Memorial Hospital, New Taipei City, Taiwan; 2 Department of Chemical Engineering and Materials Science, Yuan Ze University, Taoyuan, Taiwan; 3 Department of Pediatric Neurology, National Taiwan University Children’s Hospital, No. 7 Chung-Shan South Road, Taipei, 100 Taiwan; 4 Graduate Institute of Brain and Mind Science, National Taiwan University, Taipei, Taiwan; 5 Department of Chemical Engineering, Ming Chi University of Technology, New Taipei City, Taiwan; 6 College of Engineering, Chang Gung University, Taoyuan, Taiwan

**Keywords:** Epilepsy, ERK, Everolimus, Kainic acid, mTOR, Neuroinflammation, Rapamycin

## Abstract

**Background:**

Microglia is responsible for neuroinflammation, which may aggravate brain injury in diseases like epilepsy. Mammalian target of rapamycin (mTOR) kinase is related to microglial activation with subsequent neuroinflammation. In the present study, rapamycin and everolimus, both as mTOR inhibitors, were investigated in models of kainic acid (KA)-induced seizure and lipopolysaccharide (LPS)-induced neuroinflammation.

**Methods:**

In vitro, we treated BV2 cells with KA and LPS. In vivo, KA was used to induce seizures on postnatal day 25 in B6.129P-Cx3cr1^tm1Litt^/J mice. Rapamycin and everolimus were evaluated in their modulation of neuroinflammation detected by real-time PCR, Western blotting, and immunostaining.

**Results:**

Everolimus was significantly more effective than rapamycin in inhibiting iNOS and mTOR signaling pathways in both models of neuroinflammation (LPS) and seizure (KA). Everolimus significantly attenuated the mRNA expression of iNOS by LPS and nitrite production by KA and LPS than that by rapamycin. Only everolimus attenuated the mRNA expression of mTOR by LPS and KA treatment. In the present study, we also found that the modulation of mTOR under LPS and KA treatment was not mediated by Akt pathway but was primarily mediated by ERK phosphorylation, which was more significantly attenuated by everolimus. This inhibition of ERK phosphorylation and microglial activation in the hippocampus by everolimus was also confirmed in KA-treated mice.

**Conclusions:**

Rapamycin and everolimus can block the activation of inflammation-related molecules and attenuated the microglial activation. Everolimus had better efficacy than rapamycin, possibly mediated by the inhibition of ERK phosphorylation. Taken together, mTOR inhibitor can be a potential pharmacological target of anti-inflammation and seizure treatment.

## Background

Seizure is the clinical manifestation of abnormal, excessive, hypersynchronous discharges of a population of cortical neurons, while epilepsy is a chronic disorder characterized by recurrent unprovoked seizures [1]. Seizure could be initiated by neuronal abnormality as well as by glial activation [2, 3]. Kainic acid (KA), an agonist of kainate glutamate receptors, can cause overstimulation of glutamate receptors and subsequent neuronal excitotoxicity and neuronal death [4]. KA-induced seizure model is one of the most commonly used animal models of seizures [5].

Both clinical and experimental findings have demonstrated that inflammation may play a role in the generation and modulation of seizures and epilepsies, and using anti-inflammatory drugs, such as IL-1β blockers, has been proposed as a potential strategy for seizure therapy [6, 7]. KA administration could induce microglial activation and cytokines production, such as TNF-α, IL-1β, IL-12, and IL-18 [4]. The nucleotide-binding oligomerization domain-like receptor family pyrin domain-containing 3 (NLRP3) inflammasome triggers the transformation of procaspase-1 to caspase-1, as well as the production and secretion of mature IL-1β and IL-18 [8]. IL-1β and NLRP3 levels increased after amygdala kindling-induced status epilepticus, and inhibition of NLRP3 provided neuroprotection in rats following status epilepticus [9].

In addition to inflammation, nitric oxide (NO) plays an essential role in the epileptogenesis and excitotoxicity in the brain [10, 11]. Nitric oxide synthase (NOS) activation and NO production were observed in animal models of seizure, including the KA model [10–12]. Aminoguanidine, a selective inducible NOS (iNOS) inhibitor, attenuated KA-induced neuronal death [13], which proves the relationship between iNOS and KA-induced excitotoxicity.

The mammalian target of rapamycin (mTOR), a protein kinase, is part of two larger signaling complexes, mTORC1 and mTORC2. mTORC1, sensitive to the inhibition by rapamycin, is regulated by the upstream Akt pathway in anabolic states and by the AMPK pathway in catabolic states [14]. mTOR signaling pathway has been found to influence the immune response [15], tumorigenesis [16], brain development [17], and epilepsy [14]. Regarding immune response, mTOR is implicated in the regulation of both innate and adaptive immune responses [15]. Rapamycin (or sirolimus), the prototype mTOR inhibitor, enhanced the anti-inflammatory activities of regulatory T cells, decreased the production of proinflammatory cytokines and chemokines by macrophages and microglia, and thus attenuated secondary injury after focal ischemia in rats [18]. Several animal and human studies have shown that mTOR activation resulted in neuroexcitability, seizure, and epilepsy [14, 19], which encouraged researchers to use mTOR inhibitors in seizure therapy [14, 19, 20]. Everolimus is a second-generation rapamycin derivative. Although having a similar structure, the two drugs exhibit significant differences in their pharmacokinetic, pharmacodynamic, and toxicodynamic properties, resulting in distinct clinical profiles [21, 22]. Everolimus and rapamycin share a central macrolide structure and differ in the functional groups added at C40 [22]. The functional groups added at C40 affect their pharmacokinetics, e.g., bioavailability, half-life, and distribution [22]. Everolimus has higher potency of interacting with the mTORC 2 than rapamycin [21]. Everolimus demonstrated better ability than rapamycin in treating subependymal giant cell astrocytomas and other tuberous sclerosis (TSC) manifestations, based on more robust clinical trial experience [22]. However, to our knowledge, their efficacy in seizure treatment had never been investigated.

In this study, we used BV2 microglial cell line and B6.129P-Cx3cr1^tm1Litt^/J mice to investigate the in vitro and in vivo effects of rapamycin and everolimus on neuroinflammation. We hypothesize that their different effects on neuroinflammation may contribute to their different anti-seizure efficacies.

## Methods

### BV2 microglial cell line

BV2 cell line is the most frequently used substitute for primary microglia and has been used in studies related to neurodegenerative disorders ﻿[23]. In the present study, BV2 cells were cultured in DMEM (Corning, Manassas, VA, USA), supplemented with 10% fetal bovine serum, 1% non-essential amino acids, and 1% antibiotics (penicillin 100 U/mL, streptomycin 100 μg/mL), and were kept in an incubator at 37 °C, 5% CO_2_, and 95% relative humidity.

### Animals

B6.129P-Cx3cr1^tm1Litt^/J mice (The Jackson laboratory) possess microglia with a fluorescent protein, which expresses fluorescence when the microglia are activated by stimuli such as inflammation and damage. The mice were raised in the National Laboratory Animal Center (NLAC) in Taiwan and housed and maintained on a 12-h-on/12-h-off light/dark cycle. All of the animals were allowed free access to food and water. The maintenance of the mice and the experiments were conducted in accordance with the Guide for the Care and Use of Laboratory Animals [24] and the study was approved by the animal ethical committee of Medical College of National Taiwan University.

### Chemicals and drugs

Lipopolysaccharide (LPS), KA, minocycline, everolimus, and rapamycin were purchased from Sigma-Aldrich (St. Louis, MO, USA). The primary antibodies Akt and GAPDH used for Western blotting were purchased from Santa Cruz Biotechnology (Dallas, TX, USA) and Genetex (Irvine, CA, USA), respectively. The other primary antibodies, including ERK and phosphor-ERK, used for Western blotting were purchased from Cell Signaling (Danvers, MA, USA).

### MTT assay for cell viability

Before the nitrite assay and the qPCR assay, the MTT (3-(4,5-dimethylthiazol-2-yl)-2,5-diphenyltetrazolium bromide) assay was performed to assess whether the drugs and the combination of drugs at the specific concentrations we used affect the cell viability. BV2 cells at a concentration of 1.5 × 10^5^ cells/well were seeded into 24-well plates overnight. After treatment with different drugs for 24 h, MTT (Sigma-Aldrich, St. Louis, MO, USA) was added to each well at the final concentration of 0.5 mg/mL. After 3 h of incubation, the medium was removed and 500 μL of DMSO was added to each well. After 15 min of shaking for thorough mixing of DMSO and formazan, 200 μL of the mixture from each well was collected and placed into 96-well plates. The optical density was measured at 570 nm using a spectrophotometer. The amount of formazan formed directly correlates well with the number of live cells in the culture.

### Nitrite assay

Nitrite is a metabolite of NO, and NO production can be measured through quantification analysis of nitrite production [25]. BV2 cells at a concentration of 1.5 × 10^5^ cells/well were seeded into 24-well plates overnight. After treatment with different drugs for 24 h, 100 μL of medium from each well was collected and placed into 96-well plates, mixed with 100 μL of Griess reagent (Sigma-Aldrich, St. Louis, MO, USA) and shaken for 15 min to measure the nitrite amount. The optical density was measured at 562 nm using a spectrophotometer. The amount of nitrite in medium correlates well with the NO production by the cells.

### Real-time PCR

BV2 cells at a concentration of 4.5 × 10^5^ cells/3 mL were seeded into 40-mm dishes overnight. After treatment with different drugs for 24 h, total RNA was extracted by TRIZOL, and reverse transcribed to cDNA using the RevertAid H Minus First Strand cDNA Synthesis Kit (Thermo Scientific, Waltham, MA, USA). For real-time PCR, Maxima SYBR Green qPCR Master Mix (Thermo Scientific, Waltham, MA, USA) was used. For detecting iNOS, mTOR, NLRP3, and IL-1β level, we used the following primer sequences: iNOS, forward 5′-CTG CAT GGA ACA GTA TAA GGC AAA C-3′ and reverse 5′-CAG ACA GTT TCT GGT CGA TGT CAT GA-3′; mTOR, forward 5′-ACT GAG GAG GGA GAA CAG CA-3′ and reverse 5′-TGG CTC CAT CTG CTA GTG TG-3′; NLRP3, forward 5′-AGA GCC TAC AGT TGG GTG AAA TG-3′ and reverse 5′-CCA CGC CTA CCA GGA AAT CTC-3′; IL-1β, forward 5′-CCC TGC AGC TGG AGA GTG TGG A-3′ and reverse 5′-TGT GCT CTG CTT GTG AGG TGC TG-3′; and β-actin, forward 5′-CTA AGG CCA ACC GTG AAA AG-3′ and reverse 5′-ACC AGA GGC ATA CAG GGA CA-3′. Relative amounts of the indicated mRNA levels were determined by the 2^−ΔΔCT^ method, normalizing with β-actin levels.

### KA-induced seizures and the two-hit seizure model

The severity of seizures induced by KA can be distinguished using the modified Racine’s scale based on the abnormal behavior of mice as follows: stage I—chewing; stage II—head nodding; stage III—unilateral forelimb clonus; stage IV—bilateral forelimb clonus; stage V—bilateral forelimb clonus and falling; stage VI—running or bouncing seizure; stage VII—tonic hindlimb extension; and stage VIII—tonic hindlimb extension culminating in death [26–28]. The behavior from stage III to stage VIII can be recognized in the present mouse model. The rearing and falling behavior of stage V can be easily demonstrated in mice. Mice exhibiting at least stage V were included in this study due to microglial activation and neuronal loss in this stage [28–32]. The latency was recorded when mice first showed rearing behavior of stage V in this study. Koh et al. [33] developed a two-hit seizure model, demonstrating that “an early-life seizure permanently decreases seizure threshold and increases the susceptibility to seizure-induced cell death in adulthood”. In addition, they showed that anti-inflammatory therapy with minocycline after the initial status epilepticus blocked the epileptogenic process and mitigated the long-term damaging effects of early-life seizures [34].

Based on the 7-day protocol of Koh et al. with some modification, in this study, the postnatal day 25 (P25) mice received intra-peritoneal injection of KA 25 mg/kg on days 1 and 7. The durations from injection to stage V seizures on days 1 and 7, which were defined as latency 1 and 2, respectively, were recorded. Three hours after the seizure onset on day 1 and the following days until day 6, mice were injected with everolimus or PBS as control q.d. intraperitoneally. Mice were divided into the following three groups: KpK group, KA injection on days 1 and 7 and PBS from day 1 to day 6; KeK group, KA injection on days 1 and 7 and everolimus from day 1 to day 6; and PpP group as controls, PBS injection throughout the experiment. A 14-day protocol was followed with the same procedures: the first and second KA injection on days 1 and 14 and everolimus or PBS from day 1 to day 13.

### Quantification of microglial activation

Five to six mice were analyzed per group. Mice were sacrificed and perfused with PBS and 4% paraformaldehyde/0.1 M sodium phosphate buffer. The brains were harvested and kept in 4% paraformaldehyde/0.1 M sodium phosphate buffer for post-fixation. Before slicing the brains, they were kept in 30% sucrose/4% paraformaldehyde solution. Then the brains were cut into 30-μm horizontal slices until the hippocampus was revealed. The slices were then collected every six slices, and at least six slices were gathered for each brain. Images of the hippocampus CA1 and CA3 regions were taken by a fluorescence microscope and a camera under ×10 objective. All images were captured under identical settings. The activated microglial cells exhibited fluorescence. The number of activated microglial cells in each slice was counted within the CA1 and CA3 regions in each animal. The mean number of activated microglial cells was calculated by the Image J software. Data were expressed as the mean of activated microglial cells in CA1 or CA3 regions per slice in each animal.

### Western blotting

For in vitro experiment, the BV2 cells were seeded at a concentration of 1.5 × 10^6^ cells/10 mL into 100-mm dishes overnight. After treatment with different drugs for 24 h, the cell lysates were collected for protein analysis. For in vivo experiment, 5 to 11 mice were sacrificed 24 h after KA injection on day 7, and the hippocampi were resected and stored in liquid nitrogen. After homogenization, protein expression was analyzed by Western blotting. After electrophoresis and transfer to nitrocellulose membranes, the membranes were blocked with 5% bovine serum albumin (BSA) in PBST (PBS and 0.05% tween-20) for 30 min, incubated with primary antibodies in 5% BSA solution overnight, and then incubated with secondary antibodies in 5% BSA solution for 1 h. Signals were visualized using the ECL reagent and a chemiluminescence and fluorescence image analyzer. ImageJ software was used to subtract background and to perform densitometry.

### Statistical analysis

For in vitro and in vivo experiments, one-way ANOVA test was used to analyze cell viability, nitrite production, mRNA levels, and protein phosphorylation among the different treatment groups, with post hoc comparison by LSD test. Seizure severity at days 1 and 7 was compared using chi-square test. All data were analyzed using IBM® SPSS® Statistics software version 19.0 (IBM Inc., Somers, NY, USA). A *p* value <0.05 was considered to be statistically significant.

## Results

### No effect on cell viability under LPS and KA treatment for different drugs in BV2 cell line

The BV2 cells were treated with KA (150 μM), LPS (500 ng/mL), minocycline (1 ng/mL), everolimus (1 nM), rapamycin (1 nM), KA with minocycline, KA with everolimus, KA with rapamycin, LPS with minocycline, LPS with everolimus, and LPS with rapamycin. After 24 h of treatment, all combinations of the drugs showed no effect on the viability of BV2 cells.

### Reduction of nitrite production by everolimus under both LPS and KA treatment, while by rapamycin only under KA treatment in BV2 cell line

**Fig. 1:**
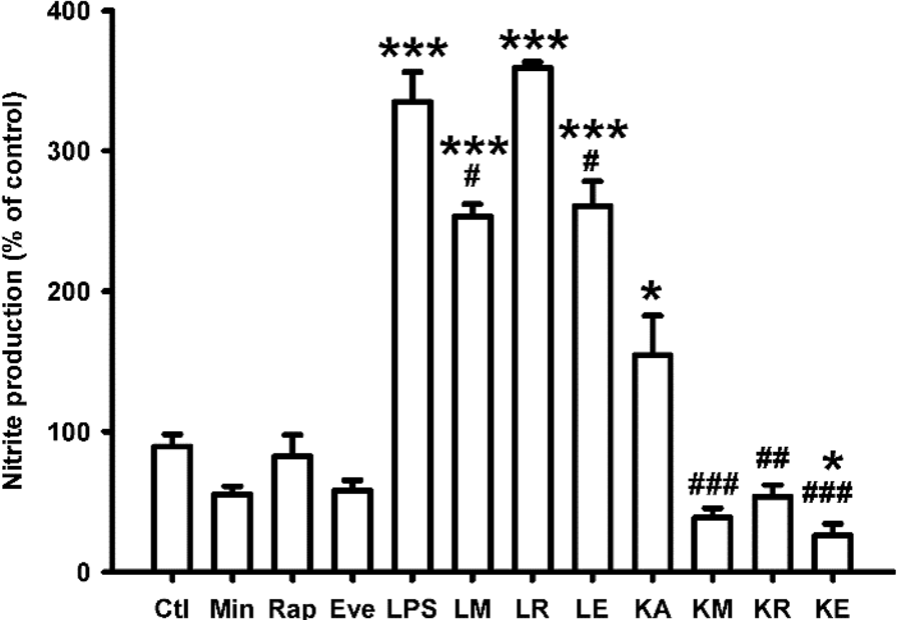
Minocycline and everolimus reduced nitrite production under both LPS and kainic acid treatment, while rapamycin only reduced nitrite production under kainic acid treatment in BV2 cell line. After treatment with different drugs for 24 hours, nitrite was assayed. Both LPS (*n* = 10) and kainic acid (KA, *n* = 4) increased nitrite production significantly. Minocycline (LM and KM groups, *n* = 5 and 4, respectively) and everolimus (LE and KE groups, *n* = 10 and 5, respectively) attenuated the increased nitrite levels under both LPS and KA treatment,. Rapamycin, however, attenuated the elevated nitrite level only under KA treatment (KR group, *n* = 5), and it had no effect on the nitrite level under LPS treatment (LR group, *n* = 5). ***p < 0.001; *p < 0.05, compared with the control group (Ctl, *n* = 17). ^###^ *p* < 0.001; ^##^ *p* < 0.01; ^#^ *p* < 0.05, compared with the LPS and KA groups, respectively. Data are presented as mean ± SEM. *Ctl* control, *Eve/E* everolimus, *KA/K* kainic acid, *LPS/L* lipopolysaccharide, *Min/M* minocycline, *Rap/R* rapamycin

As mentioned above, NO plays an essential role in the epileptogenesis and excitotoxicity in the brain [10, 11]. We therefore measured nitrite, a metabolite of NO. Previous studies have shown that both LPS and KA increased nitrite production in microglia [11, 35]. Similarly, LPS and KA significantly increased nitrite production in BV2 cell line in this study (*p* < 0.001 and *p* = 0.040, respectively) (Fig. 1). Minocycline and everolimus significantly attenuated nitrite production under both LPS and KA treatment (*p* < 0.05 and *p* < 0.001, respectively). However, rapamycin inhibited nitrite production only under KA treatment (*p* = 0.001) and did not attenuate the increased nitrite production stimulated by LPS in the BV2 cell line.

### Inhibition of iNOS mRNA production under both LPS and KA treatment by minocycline, everolimus, and rapamycin in BV2 cell line

**Fig. 2:**
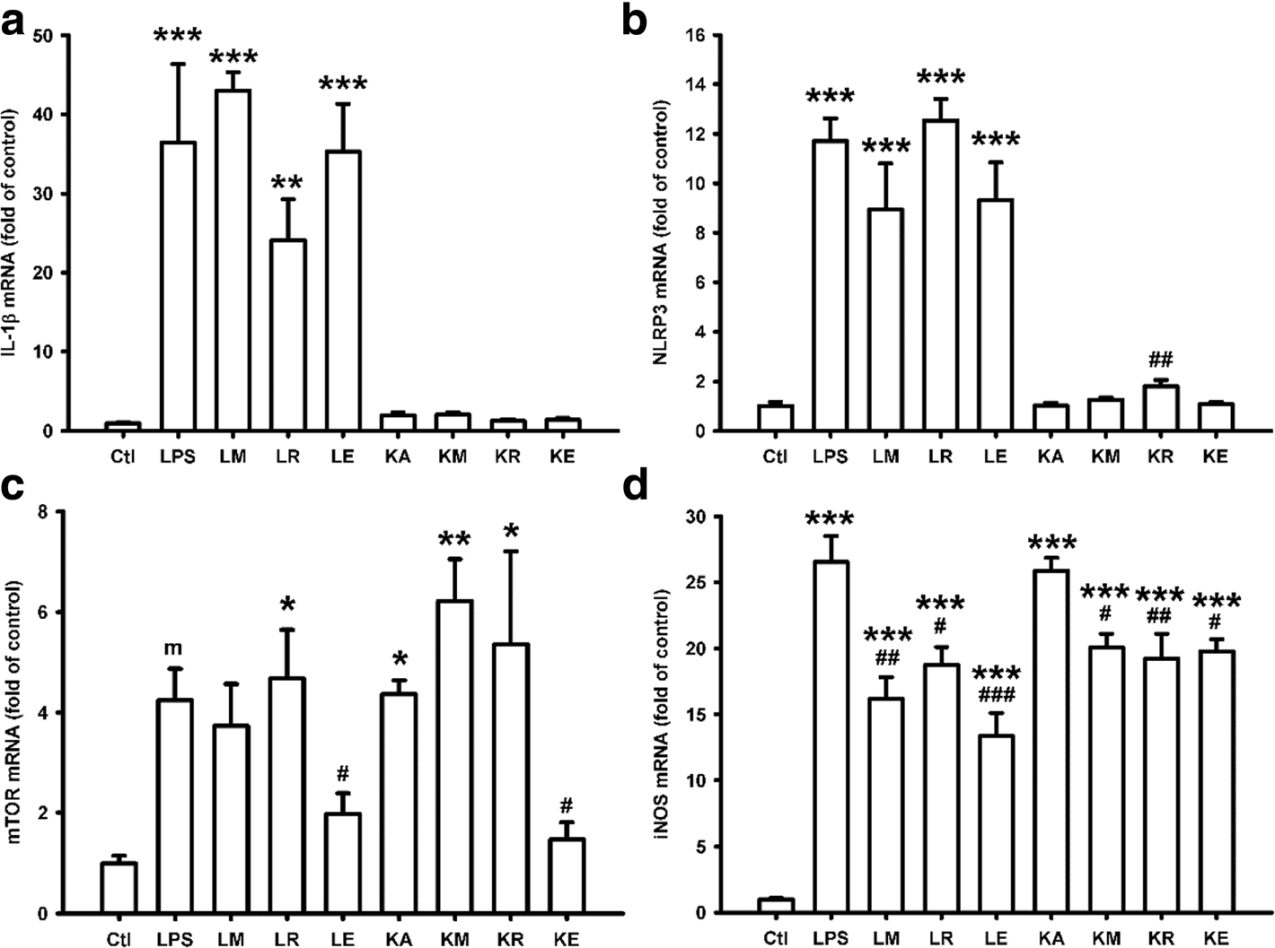
Inhibition of inflammation-related mRNAs and iNOS mRNA production under both LPS and kainic acid treatment by minocycline, everolimus, and rapamycin in BV2 cell line. Lipopolysaccharide (LPS) increased IL-1β, NLRP3, mTOR, and iNOS mRNA production (**a**–**d**), and kainic acid (KA) increased only mTOR and iNOS mRNA production (**c**, **d**). **a** Minocyclin, rapamycin, and everolimus had no effect on IL-1β mRNA production under both LPS and KA treatment. **b** Rapamycin increased NLRP3 mRNA production under KA treatment significantly, but not under LPS treatment. Minocyclin and everolimus had no effect on NLRP3 mRNA production. **c** Everolimus decreased mTOR mRNA production under both LPS and KA treatment, while minocyclin and rapamycin had no effect on it. **d** Minocyclin, rapamycin, and everolimus all attenuated iNOS mRNA production under both LPS and KA treatment. *n* = 4 for each group. ***p < 0.001; **p < 0.01; *p < 0.05; *m*: *p* < 0.1, compared with the control group (Ctl). ^###^ *p* < 0.001; ^##^ *p* < 0.01; ^#^ *p* < 0.05, compared with the LPS and KA groups. Data are presented as mean ± SEM. *Ctl* control, *E* everolimus, *KA/K* kainic acid, *LPS/L* lipopolysaccharide, *M* minocycline, *R* rapamycin

Both everolimus and rapamycin attenuated nitrite production under KA treatment, while only everolimus attenuated nitrite production under LPS treatment. We further investigated their effects on the mRNA levels of IL-1β, NLRP3, mTOR, and iNOS. LPS, a component of the outer membrane of Gram-negative bacteria, can elicit a strong immune response and has been commonly used in animal experiments of inflammation. LPS significantly increased the mRNA expression levels of IL-1β, NLRP3, and iNOS (*p* <0.001), and marginally increased expression of mTOR mRNA (*p* = 0.058) (Fig. 2). Under LPS treatment, minocycline, rapamycin, and everolimus, all inhibited the mRNA expression of iNOS (Fig. 2d), and the inhibition by everolimus was significantly better compared to that by rapamycin (*p* < 0.001). KA alone significantly increased the mRNA expression of mTOR and iNOS (*p* = 0.047 and *p* <0.001, respectively). The elevated mRNA expression of iNOS stimulated by KA was significantly attenuated by minocycline, rapamycin, and everolimus. However, only everolimus attenuated the mRNA expression of mTOR under both LPS and KA treatment (Fig. 2c, *p* = 0.021 and *p* = 0.034, respectively), and rapamycin did not inhibit mRNA expression of mTOR under both LPS and KA treatment. However, both drugs had no effect on IL-1β expression, and rapamycin increased the mRNA expression of NLRP3 under KA treatment (Fig. 2b, *p* = 0.009), which may aggravate the neuroinflammation.

### Decreased ERK phosphorylation, but not Akt phosphorylation by everolimus under both LPS and KA treatment, while that by minocycline and rapamycin only under LPS treatment in BV2 cell line

**Fig. 3:**
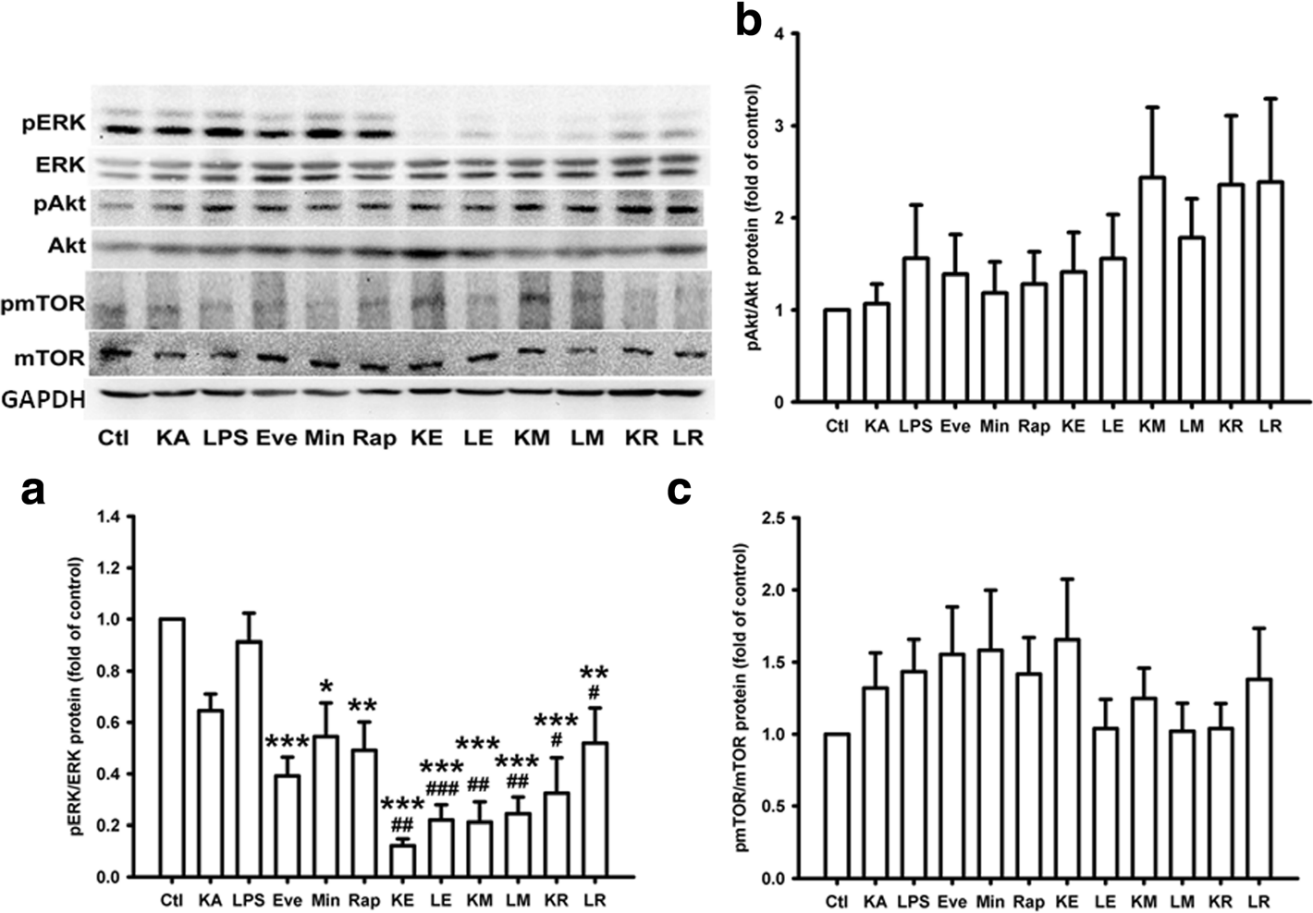
Decreased ERK phosphorylation by everolimus, minocyclin, and rapamycin under both LPS and kainic acid treatment in BV2 cell line. **a** After both LPS and kainic acid (KA) treatment for 24 h, ERK phosphorylation was attenuated significantly by everolimus (LE and KE groups), minocycline (LM and KM groups), and rapamycin (LR and KR groups), while everolimus performed best. **b**, **c** No effect on Akt and mTOR phosphorylation by everolimus, minocycline, and rapamycin was noted. *n* = 5 for each group. ***p < 0.001; **p < 0.01;*p < 0.05, compared with the control group (Ctl). ^##^ *p* < 0.01; ^#^ *p* < 0.05, compared with the LPS and KA groups. Data are presented as mean ± SEM. *Ctl* control, *Eve/E* everolimus, *KA/K* kainic acid, *LPS/L* lipopolysaccharide, *Min/M* minocycline, *Rap/R* rapamycin

Rapamycin and its analogs, e.g., everolimus, bind to FK506-binding protein 12 (FKBP12), form a ternary complex with mTORC1, and thus allosterically inhibit the functioning and downstream signaling of mTOR [36]. Interestingly, everolimus inhibited the mRNA expression of mTOR under both LPS and KA treatment in the present study, while rapamycin did not. mTOR expression is regulated by the upstream Akt pathway in anabolic states and by the AMPK pathway in catabolic states [14]. Therefore, we further investigated the influence of Akt and ERK phosphorylation by rapamycin and everolimus in the BV2 cell line. As shown in Fig 3, there was no statistically significant effect of rapamycin or everolimus treatment on Akt phosphorylation. In contrast, monotherapy with everolimus, minocycline, or rapamycin inhibited ERK phosphorylation under both LPS and KA treatment, and the effect of everolimus on the inhibition was most significant compared to those of minocycline and rapamycin (Fig. 3a, *p* < 0.001).

### Change of seizure latency after treatment with KA and everolimus

**Fig. 4:**
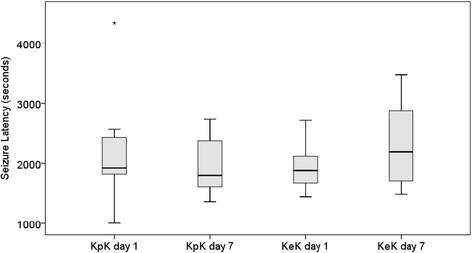
Boxplots of seizure latency to stage V in days 1 and 7 for KpK and KeK groups, showing the relative prolonged seizure latency to stage V in day 7 for KeK group

To investigate the effect of KA and everolimus treatment on the seizure latency, B6.129P-Cx3cr1^tm1Litt^/J mice, which express fluorescence when the microglial cells are activated, were used in this study. KA was administered at days 1 and 7 for the KpK group. For the KeK group, everolimus (1 mg/kg/day) was also injected daily for 7 days. The seizure staging for all mice were recorded after injection. All mice in KpK group at most reached stage V in days 1 and 7. In contrast, 4 of 12 mice in KeK group reached stage VI at day 1, while no mice in KeK group reached stage VI at day 7 (*p* = 0.047). The seizure latency to stage V after the first dose and second dose of KA was 2200 ± 874 s and 1940 ± 450 s in the KpK group (*n* = 9), and 1909 ± 363 s and 2287 ± 706 s in the KeK group (*n* = 12), respectively (*p* = 0.077) (Fig. 4). Although there was no statistical significance in seizure latency, treatment with everolimus tended to prolong the seizure latency to stage V and attenuated seizure severity.

### Significantly decreased ERK phosphorylation by everolimus in the animal model of KA-induced seizures

**Fig. 5:**
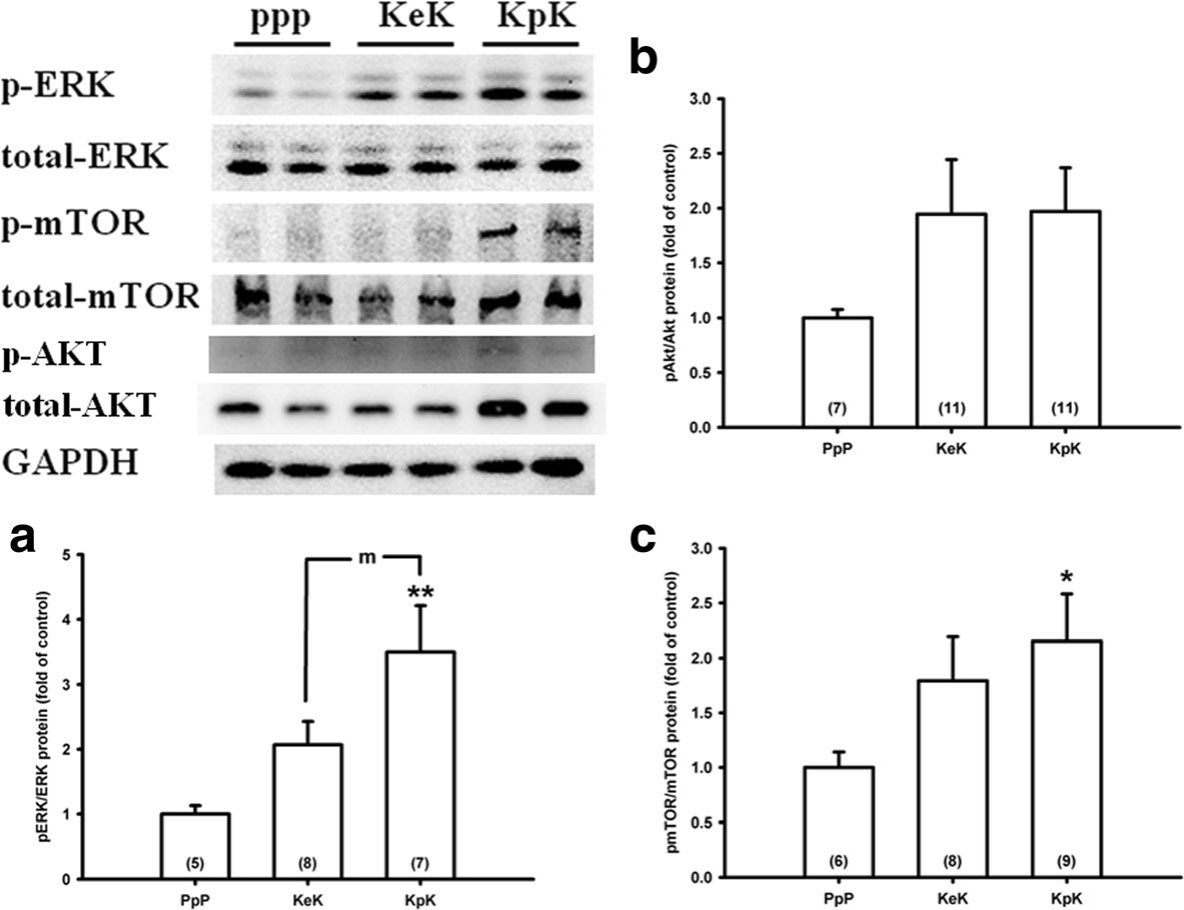
Decreased ERK phosphorylation by everolimus significantly in the animal model of kainic acid-induced seizures. B6.129P-Cx3cr1^tm1Litt^/J mice received kainic acid (KA) i.p. injection on day 1 and day 7 (two-hit seizure model), and received i.p. injection of PBS (KpK group) or everolimus (KeK group) during day 1 to day 6. Sham mice received PBS during day 1 to day 7 (PpP group). Twenty-four hours after KA injection on day 7, mice were sacrificed, the hippocampus was resected, and the protein expression was analyzed by Western blotting. **a** KA injection twice significantly increased ERK phosphorylation, which was attenuated by everolimus. **b**, **c** Everolimus did not decrease Akt and mTOR phosphorylation after repeated KA injection. *Bars* depict mean ± S.E.M. The number of mice used in each experiment was shown in the bottom of each bar figure. *p = 0.034 and **p = 0.004, compared with the sham group; m: *p* = 0.048, compared between KeK and KpK groups

To support our in vitro finding, we investigated the effect of everolimus on Akt and ERK phosphorylation by applying the two-hit seizure model of KA in mice [33]. The two-hit seizure model of KA i.p. injection at days 1 and 7 significantly increased ERK phosphorylation (*p* = 0.004) and mTOR phosphorylation (*p* = 0.034) in the hippocampus of mice in the KpK group (Fig. 5 a, c). Compared with the KpK group, everolimus significantly inhibited ERK phosphorylation similar to the results of the in vitro studies (Fig. 5a, KeK group, *p* = 0.048). However, there was no significant difference in ERK phosphorylation in control group and KeK group (*p* = 0.167). In contrast, the two-hit seizure model of KA injection did not increase Akt phosphorylation significantly (Fig. 5b, *p* = 0.135).

### Treatment with everolimus decreased the microglial activation in the hippocampus under KA treatment

To investigate the effect of everolimus treatment on microglial activation in the hippocampus (both CA1 and CA3 regions), we further counted the activated microglial cells in the KpK and KeK groups at day 15 after treatment with KA for two times. We found that the number of activated microglial cells in the CA1 and CA3 regions was statistically significantly higher in the KpK groups than in the KeK group. The mean number of activated microglial cells in CA1 and CA3 regions per slice in the KpK and KeK groups was 51.8 ± 22.1 vs. 15.1 ± 5.1 in CA1 (*p* = 0.009) and 35.7 ± 6.2 vs. 9.5 ± 2.2 in CA3 regions, respectively (*p* < 0.001). This result indicated that everolimus can significantly downregulate the activation of microglial cells in the hippocampus of mice with KA-induced seizures.

## Discussion

**Fig. 6:**
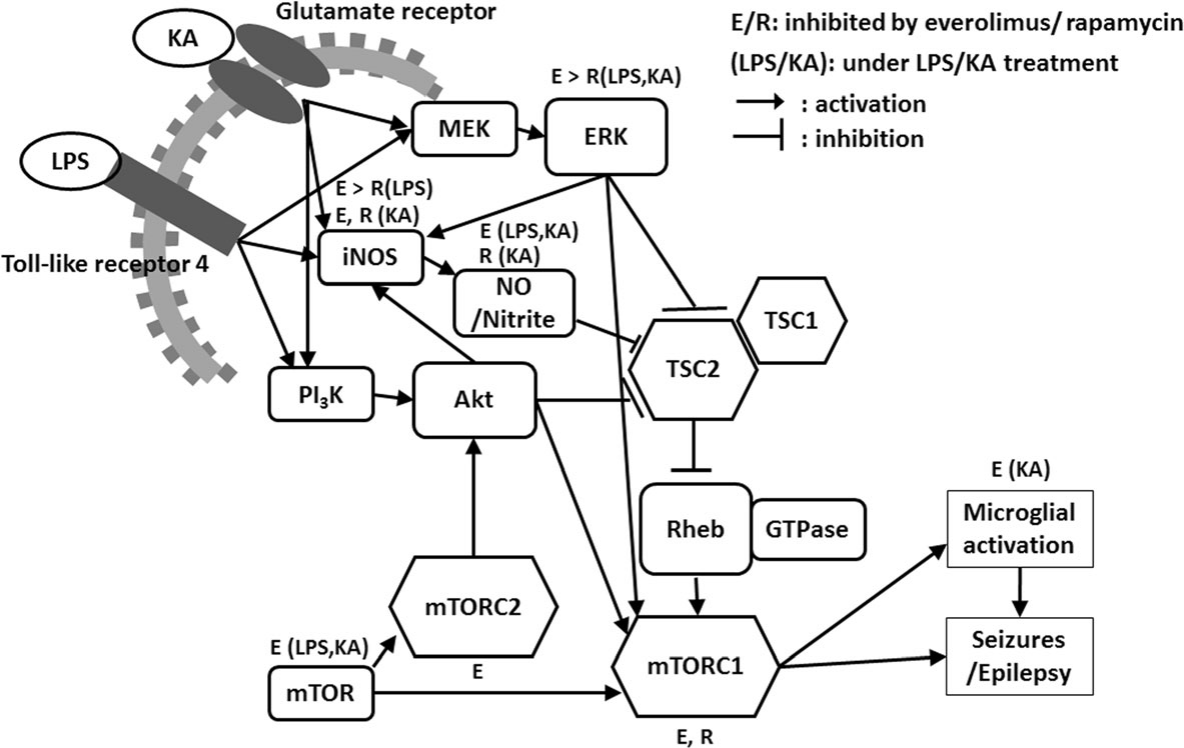
A summary of the effects of rapamycin and everolimus on signaling pathways involved in neuroinflammation and seizure. The direct inhibition of mTORC1 and mTORC2 by rapamycin and everolimus is from previous studies. The other effects of rapamycin and everolimus depicted in this figure are based on this study. *E* everolimus, *ERK* extracellular signal-regulated kinases, *iNOS* inducible nitric oxide synthase, *KA* kainic acid, *LPS* lipopolysaccharide, *mTORC1/2* mechanistic target of rapamycin complex 1/2, *NO* nitric oxide, *R* rapamycin, *Rheb* Ras homolog-enriched in brain, *TSC1/2* tuberous sclerosis complex 1/2

Several animal and human studies have demonstrated that mTOR activation can result in neuroexcitability, seizure, and epilepsy [14, 19]. Therefore, mTOR inhibitors have been applied in seizure therapy. Although both rapamycin and everolimus are mTOR inhibitors, the present study showed that both drugs had differential effects on nitrite production, mRNA expression of iNOS and mTOR, and ERK phosphorylation (Fig. 6). Both drugs had no effect on IL-1β expression while rapamycin increased NLRP3 expression, which may aggravate the neuroinflammation. Everolimus was significantly more effective than rapamycin in inhibiting iNOS and mTOR signaling pathways in both models of neuroinflammation (LPS) and seizure (KA). In the iNOS pathway, everolimus attenuated the iNOS mRNA expression stimulated by LPS and nitrite production by KA and LPS more significantly than rapamycin. In the mTOR pathway, only everolimus attenuated the mTOR mRNA expression induced by LPS and KA treatment. We also found that the modulation of mTOR under LPS and KA treatment was not mediated by Akt pathway but may be primarily mediated by ERK phosphorylation, which was attenuated more significantly by everolimus. Everolimus was also shown to inhibit ERK phosphorylation and microglial activation in the hippocampus of KA-treated mice.

Although not involved in regulating KA seizure generation and propagation, NO has been shown to be involved in status epilepticus-induced neuronal degeneration [11]. Rapamycin has been shown to reduce the mRNA levels of iNOS in the astrocytes under treatment with cytokines or LPS plus INFg [37]. Everolimus has also been shown to affect NOS activity and NOS2 expression, thereby reducing microglial proliferation [38]. The present study demonstrated that both rapamycin and everolimus can decrease iNOS mRNA and nitrite production after LPS or KA treatment in the microglia, suggesting that the neuroprotective role of mTOR inhibitors may partially arise from iNOS inhibition. However, our data also showed that everolimus was significantly more effective than rapamycin in the inhibition of iNOS and nitrite production.

The reason why rapamycin increased NLRP3 mRNA in the present study was not clear. NO has been reported to suppress NLRP3 inflammasome activation under LPS treatment [39]. In our study under KA treatment, rapamycin inhibited iNOS mRNA and NO/nitrite production, which may contribute to the increase of NLRP3 mRNA (Fig. 2). However, everolimus also inhibited iNOS mRNA and NO/nitrite production without increase of NLRP3 mRNA, which needs further investigation.

mTOR is part of two larger signaling complexes, mTORC1 and mTORC2. The primary pharmacodynamic effect of mTOR inhibitors is selective binding to FKBP12 and subsequent association with and inhibition of mTORC 1 [22]. Everolimus exhibited higher potency of interacting with mTORC 2 than rapamycin [21] and was shown to be better than rapamycin in treating subependymal giant cell astrocytomas and other TSC manifestations [22]. Interestingly, our study also showed that everolimus decreased the mRNA levels of mTOR under LPS and KA treatment compared with rapamycin. This finding may explain the higher potency of everolimus in inhibiting mTORC2 than that of rapamycin.

mTOR is regulated by the upstream Akt pathway and the AMPK pathway [14]. Inhibition of ERK phosphorylation by everolimus observed in the present study is consistent with an earlier study [40]. In a previous study of anti-HLA antibody-mediated endothelial cell signaling, everolimus was shown to be more effective in inhibiting mTORC2 and thus more effective in preventing Akt phosphorylation and ERK phosphorylation, an ability that rapamycin lacked [40]. ERK pathway plays a well-known role in neuroinflammation and neurodegeneration [41]. Therefore, everolimus may play a protective role in neuroinflammation via inhibiting ERK phosphorylation. Interestingly, in *N*-methyl-d-aspartic acid-induced retinal neurotoxicity in rats, the protective effect of everolimus was mediated partially by the activation of ERK pathway [42]. Nevertheless, both anti-inflammation and neuroprotection by everolimus are beneficial.

Our study showed that everolimus reduced neuroinflammation more effectively than rapamycin. mTORC1 and mTORC2 play different roles in inflammation [43, 44]. mTORC2 exerts a pro-inflammatory effect, while mTORC1 exerts some anti-inflammatory effect [44]. Therefore, everolimus, by inhibiting mTORC2, may be more effective than rapamycin in inhibiting neuroinflammation. Similarly, a recent study showed that a dual mTORC1 and mTORC2 inhibitor was more effective against neuroinflammation than rapamycin [45]. In addition, as shown in the present study and previous studies, everolimus, but not rapamycin, can inhibit ERK phosphorylation [21]. This inhibition of ERK pathway may augment the anti-inflammatory activity of everolimus.

Other mechanisms except for attenuating neuroinflammation may also play a role in anti-seizure activity of mTOR inhibitors. Activation of the mTOR pathway may trigger several downstream cellular and molecular events in brain leading to increased neuronal excitability and seizure generation. mTOR pathway is also implicated in epileptogenesis, especially mossy fiber sprouting [46]. Furthermore, the mTOR pathway may be involved in anti-seizure effects of the ketogenic diet and has a close link with nutrient signaling [47]. Therefore, the anti-seizure effect of mTOR inhibitors may arise from multiple mechanisms, and attenuation of neuroinflammation is one of the important mechanisms.

In this study, we also found that mice treated with everolimus following the initial seizure tended to prolong the seizure latency at second KA-induced seizure. Microglial activation with production of proinflammatory cytokines plays important roles in seizure generation [48, 49]. Previous study had shown that KA-induced exaggerated microglial response may increase the susceptibility to the second seizure later in life and produce CNS injury [34, 48, 49]. Therefore, the inhibition of seizure-induced microglial activation may prolong the seizure latency and attenuate the seizure-related CNS damage [50, 51].

## Conclusions

In this study, a direct comparison of rapamycin and everolimus in both cell and animal models of neuroinflammation and seizure was made. Everolimus showed greater inhibition of iNOS mRNA production, nitrite production, and mTOR mRNA production than rapamycin, which may partly arise from the inhibition of ERK phosphorylation. mTOR as a target of anti-epileptic therapy may be a potential pharmacological target of reducing neuroinflammation and deserves more application in the future.

## Abbreviations

BSA: Bovine serum albumin
KA: Kainic acid
LPS: Lipopolysaccharide
mTOR: Mammalian target of rapamycin
NLRP-3: Nucleotide binding and oligomerization domain-like receptor family pyrin domain-containing 3
NO: Nitric oxide
NOS: Nitric oxide synthase
